# The effect of Vdr gene ablation on global gene expression in the mouse placenta

**DOI:** 10.1016/j.gdata.2015.08.022

**Published:** 2015-08-18

**Authors:** Sam Buckberry, Fleur Spronk, Rebecca L. Wilson, Jessica A. Laurence, Tina Bianco-Miotto, Shalem Leemaqz, Sean O'Leary, Paul H. Anderson, Claire T. Roberts

**Affiliations:** a Robinson Research Institute, The University of Adelaide, School of Paediatrics and Reproductive Health, Adelaide, Australia; b School of Agriculture, Food and Wine, University of Adelaide, Adelaide, Australia; c School of Pharmacy and Medical Sciences, Division of Health Sciences, University of South Australia, Adelaide, Australia

**Keywords:** Vitamin D, Placenta, Gene expression, VDR

## Abstract

The effects of vitamin D are mediated through the vitamin D receptor (VDR), a predominantly nuclear receptor, expressed in numerous tissues including the placenta. VDR and the retinoid X receptor (RXR) form a dimer complex which binds to genomic vitamin D responsive elements located primarily in promoter regions and recruit cell-specific transcription factor complexes which regulate the expression of numerous genes. To investigate the role of VDR on regulating placental gene expression, mice heterozygous (+/−) for an ablated *Vdr* allele (C57Bl6 strain B6.129S4-VDRtm1Mbd/J, Jackson Laboratory) were mated to generate Vdr^+/+^, Vdr^+/−^ and Vdr ^−/−^ fetuses and placental samples were collected at day 18.5 of pregnancy. RNA was isolated from placental tissue with global gene expression measured using Affymetrix Mouse Gene 2.1 ST Arrays to assess the effects of VDR on global gene expression in the placenta. All raw array data are deposited in Gene Expression Omnibus (GEO) under accession GSE61583.

**Table:** 

Specifications	
Organism/cell line/tissue	Global *Vdr* ablated C57Bl6 (strain B6.129S4-VDRtm1Mbd/J, Jackson Laboratory JAX Mice Services) mouse placenta tissue collected at day 18.5 of pregnancy.
Sex	Male and female
Sequencer or array type	Affymetrix Mouse Gene 2.1 ST Array
Data format	Raw CEL files
Experimental factors	3 genotype groups, Vdr knockout (−/−), Vdr heterozygous (+/−) and wild-type (+/+).
Experimental features	Vdr heterozygous males and females were mated to generate Vdr knockout, heterozygous and wild-type offspring. At day 18.5 of pregnancy, mouse placentas were collected and RNA was extracted and assayed on Affymetrix MoGene 2.1 ST arrays to measure the effect of Vdr on global gene expression.
Consent	Data is publicly available and open for re-use given appropriate citation.
Sample source location	Adelaide, Australia

## 1 Direct link to deposited data

http://www.ncbi.nlm.nih.gov/geo/query/acc.cgi?acc=GSE61583.

## 2 Experimental design, materials and methods

Male and female mice heterozygous for the Vdr allele (Vdr knockout strain B6.129S4-Vdrtm1Mbd/J — The Jackson Laboratory) were mated at 10–12 weeks of age to generate offspring of the three genotype combinations: Vdr knockout, Vdr heterozygotes and Wild-type. Twelve *Vdr*^+/−^ females were mated at 10–12 weeks of age with *Vdr*^+/−^ males. At day 18.5, females were euthanised and placental tissue was collected and bisected mid-sagittally with half stored in RNAlater and at − 80 °C. All mice were raised on a standard chow diet.

To isolate RNA, placental tissue was homogenised using a Powerlyzer with ceramic 1.4 mm beads before extraction using TRIzol (Invitrogen) following the manufacturer's instructions. To determine fetal Vdr genotypes and sex, DNA was extracted from fetal tails and genotyped by PCR as described in reference [1].

Biotinylated cRNA was prepared according to the standard Affymetrix protocol from 250 ng total RNA following the Manual Target Preparation Guidelines for GeneChip® Whole Transcript (WT) Expression Arrays. 3.5 μg of fragmented and labeled single-stranded cRNA was hybridized on Affymetrix MoGene 2.1 ST arrays and washed and stained following the Manual Target Preparation Guidelines for GeneChip® Whole Transcript (WT) Expression Arrays. Arrays were scanned using the Affymetrix GeneChip scanner by the Ramaciotti Centre for Genomics, Sydney, Australia.

**Fig. 1:**
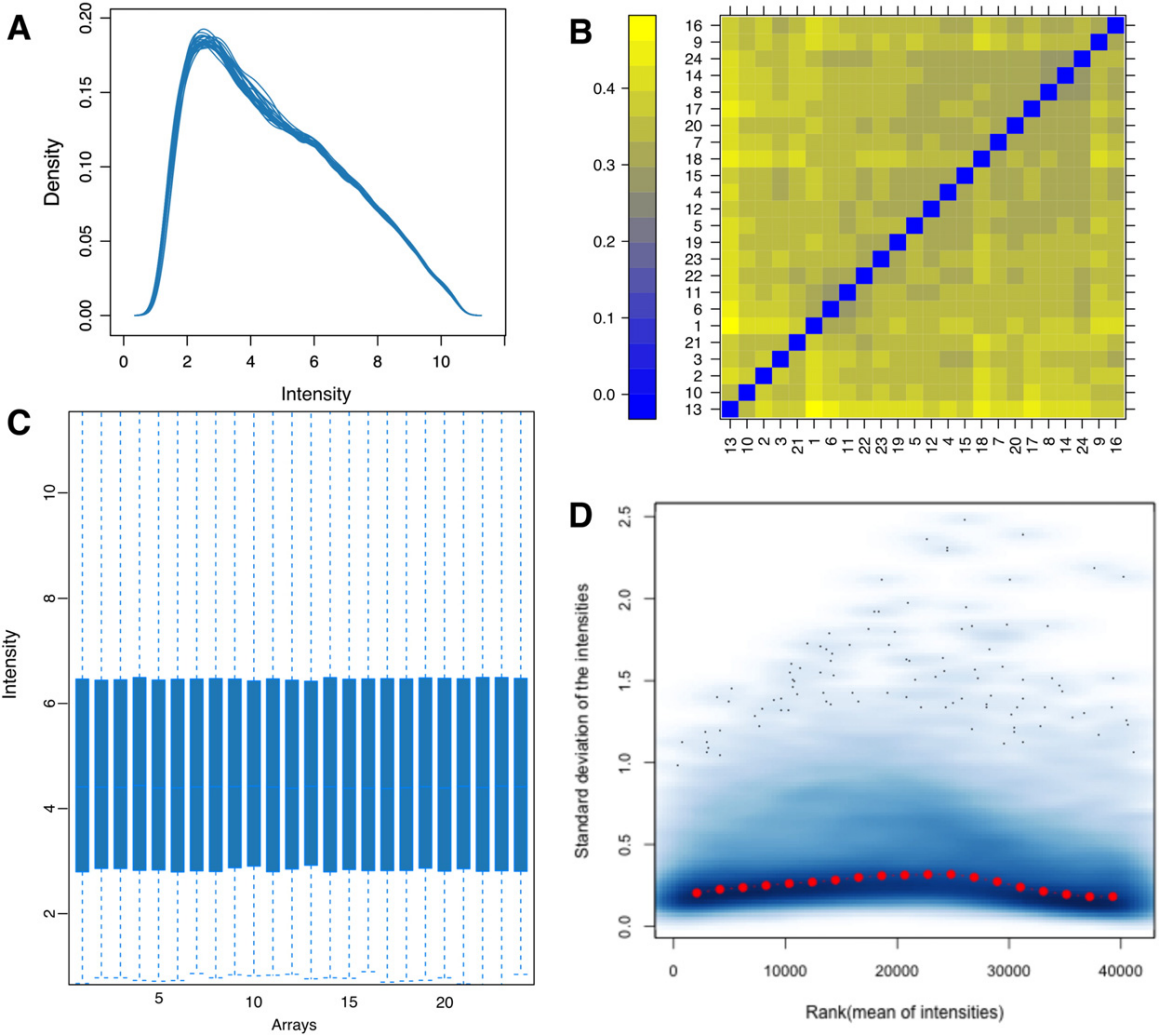
A. Density estimate distributions of un-normalised data. B. False colour heat map of distances between arrays. C. Boxplots representing summaries of the signal intensity distributions of the arrays, with each box corresponding to one array. D. Density plot showing the standard deviation of the intensities across arrays on the *y*-axis versus the rank of their mean on the *x*-axis. The red dots show the running median of the standard deviation.

Microarray quality control was carried out on the raw un-normalised using the ArrayQualityMetrics R package [2], which showed that there were no significant outlier samples and the data appeared to be of good quality (Fig. 1).

The results of this study, along with detailed experimental design and discussion are presented in reference [1]. This work was supported by a National Health and Medical Research Council Project grant awarded to CTR and PHA (GNT1020754).
